# Prognostic impact of HbA1c variability on long-term outcomes in patients with heart failure and type 2 diabetes mellitus

**DOI:** 10.1186/s12933-018-0739-3

**Published:** 2018-06-30

**Authors:** Jun Gu, Jian-an Pan, Yu-qi Fan, Hui-li Zhang, Jun-feng Zhang, Chang-qian Wang

**Affiliations:** Department of Cardiology, Shanghai Ninth People’s Hospital, Shanghai Jiaotong University School of Medicine, No. 639 Zhizaoju Road, Shanghai, 200011 People’s Republic of China

**Keywords:** Heart failure, Type 2 diabetes mellitus, Hemoglobin A1c variability, Mortality, Hospitalization

## Abstract

**Background:**

The prognostic impact of long-term glycemic variability on clinical outcomes in patients with heart failure (HF) and type 2 diabetes mellitus (T2DM) remains unclear. We determined and compared hemoglobin A1c (HbA1c) variability and clinical outcomes for patients with HF with preserved ejection fraction (HFpEF), HF with mid-range ejection fraction (HFmrEF) and HF with reduced ejection fraction (HFrEF) in a prospective longitudinal study.

**Methods:**

Patients with HF and T2DM, undergone 3 or more HbA1c determinations during the first 18 months, were then followed for 42 months. The primary outcome was death from any cause. Secondary outcome was composite endpoints with death and HF hospitalization. Cox proportional hazards models were used to compare outcomes for patients with HFpEF, HFmrEF and HFrEF.

**Results:**

Of 902 patients enrolled, 32.2% had HFpEF, 14.5% HFmrEF, and 53.3% HFrEF. During 42 months of follow-up, 270 (29.9%) patients died and 545 (60.4%) patients experienced composite endpoints of death and HF readmission. The risk of all-cause death or composite endpoints was lower for HFpEF than HFrEF. Moreover, higher HbA1c variability was associated with higher all-cause mortality or composite endpoints and HbA1c variability was an independent predictor of all-cause mortality or composite endpoints, regardless of EF.

**Conclusions:**

This prospective longitudinal study showed that the all-cause death and composite events was lower for HFpEF than HFrEF. HbA1c variability was independently and similarly predictive of death or combined endpoints in the three HF phenotypes.

**Electronic supplementary material:**

The online version of this article (10.1186/s12933-018-0739-3) contains supplementary material, which is available to authorized users.

## Introduction

Heart failure (HF), including HF with preserved ejection fraction (HFpEF), HF with mid-range ejection fraction (HFmrEF) and HF with reduced ejection fraction (HFrEF), is a progressive disease with high mortality and morbidity, and its prevalence is rising in the aging population [1]. And diabetes mellitus (DM) is a frequent comorbidity of HF, that poses an enormous medical, societal and financial burden worldwide, with more than 40% of patients with HF having DM as a discharge diagnosis [1, 2]. A number of studies have demonstrated that DM significantly increases the risk of recurrent HF hospitalizations and the duration of hospital stay in HF patients, and it is associated with a significantly higher mortality compared with those without DM [2, 3].

Glycemic variability is a general denomination to several measures of short-term or long-term fluctuations in glucose level. Short-term glycemic variability refers to within-day or between-days glycemic fluctuations, and is usually measured by continuous glucose monitoring. Long-term glycemic variability refers to glycemic fluctuations over months to years and is generally measured by visit-to-visit variability in either hemoglobin A1c (HbA1c) or fasting glucose. A limited number of literatures have indicated that both higher short-term variability and long-term glycemic variability increase the risk of cardiovascular disease (CVD) morbidity and mortality in diabetic patients [4–7]. Our recent studies showed that long-term glycemic variability was associated with the new-onset atrial fibrillation (AF) and HFpEF progression in patients with type 2 DM (T2DM) [8, 9]. However, little is known about the prognostic importance of long-term glycemic variability in patients with HF and T2DM.

The aim of this study was to evaluate the prognostic value of long-term HbA1c variability for all-cause mortality as well as combined endpoints of death or HF readmission in our HF comorbidity with T2DM cohort study.

## Methods

### Study design and population

We conducted a prospective longitudinal study of adults with HF from Shanghai Ninth People’s Hospital. Patients were those over age 18 years with a clinical diagnosis of HF and T2DM, according to the attending physician between January 2008 and March 2013. Recruitment occurred either where the patient was in hospital for a primary diagnosis of HF (assessment was done following stabilization of the acute HF) or in the out-patient setting within 6 months of an episode of decompensated HF (requiring hospitalization or treatment in an out-patient setting). Enrolled patients had experienced at least 3 HbA1c measurements during the first 18 months (baseline HbA1c variability), and were then followed for 42 months. Exclusion criteria included severe valve disease, transient acute pulmonary edema in the context of primary acute coronary syndrome, end-stage renal failure (estimated glomerular filtration rate, eGFR < 30 mL/min/1.73 m^2^), specific HF subgroups (including constrictive pericarditis, congenital heart disease, hypertrophic cardiomyopathy, cardiac amyloid, and chemotherapy-associated cardiomyopathy), isolated right HF, life-threatening comorbidity with life expectancy < 1 year. Patients who experienced all-cause death or HF hospitalization during the period of baseline HbA1c variability (the first 18 months) were also excluded. The study protocol was approved by the local ethics committee and informed consent was obtained from all patients.

### Long-term glycemic variability measurements

The intra individual mean (HbA1c-mean) was calculated from the mean value of serially measured HbA1c in each participant. HbA1c variability was measured as the standard deviation of serial HbA1c measurements (HbA1c-SD), the coefficient of variation of HbA1c (HbA1c-CV) was used to correct for the mean. On account of the lack of standard cutoff value for HbA1c variability indices at present, we categorized the subjects into two groups (high group and low group) on the basis of the median value of each HbA1c variability index as we previously described [8, 9].

### Echocardiography

Transthoracic echocardiography was performed using the Cardiovascular Ultrasound System (GE VIVIDT, GE Healthcare, LaMarquel, TX, USA) as we previously described [10–12]. Briefly, the frequency of the ultrasonic probe was 2.5 MHz. The cardiac structure and function were assessed from the M-mode guided by two-dimensional imaging to obtain the echocardiographic variables. The average of three measurements was used for each variable. Left ventricular (LV) volumes were measured using the biplane method of disks, and LVEF was determined using biplane modified Simpson’s measurements. HFpEF was defined as LVEF ≥ 50% or qualitatively ‘normal’ EF; HFmrEF as LVEF 40–49%, and HFrEF as LVEF < 40% according to 2016 ESC guidelines [1]. Tissue Doppler was performed in the apical four chamber view to obtain mitral annulus velocities. The sample was placed at the junction of the LV lateral wall with the mitral annulus and at the junction of the posterior interventricular septum with the mitral annulus; then, the early (e′) diastolic mitral annulus velocities and the E/e′ ratio were determined.

### Endpoints

The primary outcome was defined as all-cause mortality. The secondary outcome was composite endpoints of death or HF hospitalization.

### Follow-up

Most of the patients visited our out-patient clinic at least every 3 months. However, if the patients did not appear at their scheduled clinic, they were interviewed by telephone annually. Information regarding the primary and secondary outcomes was documented in chart records and via telephone interviews. For each patient, the time to death or cardiovascular events was calculated from the initial date of follow-up to the date that the primary or secondary outcome occurred.

### Statistical analysis

Statistical analysis was performed using SPSS Statistical Software, version 22.0 (SPSS Inc., Chicago, IL, USA). Arithmetic means ± standard deviations were calculated for quantitative variables, while qualitative variables were given as frequency and percentage (%). For quantitative variable analysis, the *t* test was used. A two-sided Chi square test was used to compare qualitative variables. Differences in clinical endpoints between high and low HbA1c variability stratified by HF phenotype were tested with Chi squared test. Cox proportional hazards regression model was used to explore the association between risk factors and the risk of all-cause mortality or composite endpoints. All predictors with a significance of P ≤ 0.10 in the uni-variable analysis and forced inclusion variables that were considered as important predictors of clinical endpoints were entered into the multivariable model. Hazard ratios (HR) and corresponding 95% confidence intervals (CIs) are reported. Freedom from occurrence of all-cause mortality or composite endpoints at 42 months was analyzed with Kaplan–Meier statistics, with difference between groups assessed using the log-rank test. All values were two-tailed, and a P value < 0.05 was considered statistically significant.

## Results

### Screening, recruitment and baseline clinical characteristics

**Fig. 1:**
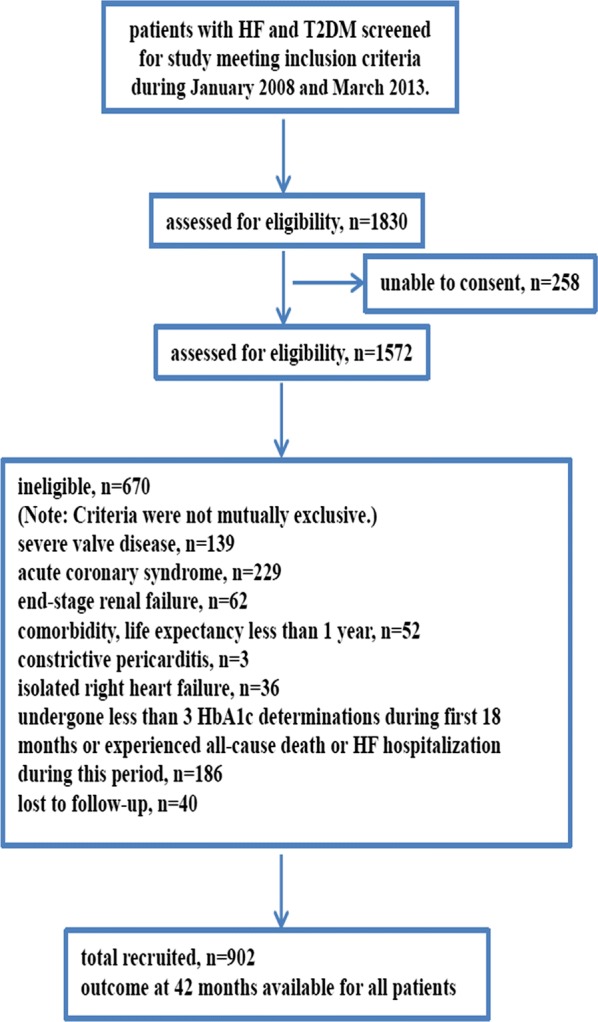
Flowchart of the study protocol

A total of 1830 patients were potentially eligible for the study, 258 were unable to provide informed consent, and a further 670 met one or more of the study exclusion criteria, leaving 902 patients included in the study (Fig. 1). Overall, mean age was 69.3 ± 7.5 years, and 296 (32.8%) were women (Table 1). Compared to patients with HFrEF, those with HFpEF were older (mean age 70.6 years vs. 68.4 years), more often female (41.0% vs. 27.4%), more likely to have a history of hypertension (73.8% vs. 63.4%) and AF (39.0% vs. 29.9%) and less often ischemic HF (40.0% vs. 49.9%). HF medications were commonly used at the time of the baseline assessment, with 724 (80.3%) of the whole group receiving an angiotensin converting enzyme inhibitor (ACEI)/angiotensin receptor blocker (ARB) and 683 (75.7%) receiving a beta-blocker, however, more HFrEF patients received ACEI/ARB (70.3% in HFpEF, 85.2% in HFrEF) or beta-blocker (69.0% in HFpEF, 81.7% in HFrEF) therapy. Furthermore, spironolactone was more frequently prescribed in HFrEF patients (24.8% in HFpEF, 38.7% in HFrEF). Functional status (New York Heart Association, NYHA class) was similar in HFpEF and HFrEF. The clinical characteristics of the patients with HFmrEF were similar to the HFpEF group, except for systolic blood pressure (SBP), which was similar to HFrEF (Table 1). In regard to the echocardiographic findings, patients had a relative lower E/e′ ratio and smaller left atrium diameter (LAD) in HFpEF. And B-type natriuretic peptide (BNP) in HFrEF was higher compared with HFpEF or HFmrEF. The median value of HbA1c-SD and HbA1c-CV were 0.6722 and 9.1896%, respectively. All patients were followed-up for 42 months.

**Table 1:** Baseline characteristics

	HFpEF (LVEF ≥ 50%)	HFmrEF (LVEF 40–49%)	HFrEF (LVEF < 40%)	P value
n	290 (32.2)	131 (14.5)	481 (53.3)	
Age (years)	70.6 ± 6.7	69.7 ± 6.0	68.4 ± 8.2	< 0.001
Women (gender)	119 (41.0)	45 (34.4)	132 (27.4)	< 0.001
Medical history				
Ischemic HF	116 (40.0)	55 (42.0)	240 (49.9)	0.019
Prior PCI	64 (22.1)	26 (19.8)	111 (23.1)	0.729
Prior CABG	17 (5.9)	6 (4.6)	33 (6.9)	0.604
Hypertension	214 (73.8)	94 (71.8)	305 (63.4)	0.007
Duration of T2DM (years)	8.3 ± 2.7	8.3 ± 2.4	8.3 ± 2.4	0.875
Atrial fibrillation	113 (39.0)	52 (39.7)	144 (29.9)	0.014
Stroke	32 (11.0)	12 (9.2)	56 (11.6)	0.724
COPD	32 (11.0)	11 (8.4)	52 (10.8)	0.687
Smoking	81 (27.9)	39 (29.8)	149 (31.0)	0.670
Dyslipidemia	82 (28.3)	40 (30.5)	139 (28.9)	0.894
HF device-therapies				
ICD	5 (1.7)	1 (0.7)	8 (1.7)	0.868
CRT-P	0	0	4 (0.8)	0.284
CRT-D	0	0	4 (0.8)	0.284
Medications				
ACEI/ARB	204 (70.3)	110 (84.0)	410 (85.2)	< 0.001
Beta-blocker	200 (69.0)	90 (68.7)	393 (81.7)	< 0.001
Diuretics	163 (56.2)	83 (63.4)	299 (62.2)	0.196
Spironolactone	75 (24.8)	33 (25.2)	186 (38.7)	< 0.001
Anticoagulant	29 (10.0)	14 (10.7)	45 (9.4)	0.882
Antiplatelet	146 (50.3)	58 (44.3)	259 (53.8)	0.142
Statin	107 (36.9)	47 (35.9)	188 (39.1)	0.728
Oral anti-diabetic drugs	175 (60.3)	86 (65.6)	326 (67.8)	0.110
Sulfonylurea	116 (40.0)	56 (42.7)	216 (44.9)	0.410
Glinides	26 (9.0)	11 (8.4)	32 (6.6)	0.475
Biguainde	64 (22.1)	26 (19.8)	92 (19.1)	0.641
α-GI	42 (14.5)	21 (16.0)	56 (11.6)	0.309
DPP-4 inhibitor	17 (5.9)	7 (5.3)	29 (6.0)	0.957
Insulin	83 (28.6)	35 (26.7)	113 (23.5)	0.273
Clinical status				
NYHA class, in Classes I–IV	26/110/138/16	21/43/59/8	81/182/200/18	0.052
Heart rate (bpm)	80.1 ± 8.9	78.9 ± 8.5	80.3 ± 10.3	0.356
Systolic BP (mmHg)	132.0 ± 11.9	128.6 ± 12.1	128.9 ± 14.8	0.006
Diastolic BP (mmHg)	78.5 ± 9.0	76.7 ± 7.8	77.7 ± 7.8	0.128
Laboratory variables				
eGFR (mL/min/1.73 m^2^)	61.6 ± 9.5	61.7 ± 9.5	60.3 ± 9.0	0.104
Haemoglobin (g/dL)	11.8 ± 1.4	11.9 ± 1.2	12.0 ± 1.2	0.175
BNP (pg/mL)	772.0 ± 309.6	804.9 ± 306.1	912.0 ± 489.5	< 0.001
Number of HbA1c measurements	10.4 ± 1.9	10.6 ± 1.9	10.7 ± 2.0	0.108
Baseline HbA1c (%)	7.2 ± 0.6	7.2 ± 0.5	7.2 ± 0.6	0.307
HbA1c-mean (%)	7.2 ± 0.6	7.2 ± 0.5	7.3 ± 0.6	0.103
HbA1c-SD (%)	0.66 ± 0.09	0.65 ± 0.08	0.67 ± 0.08	0.061
HbA1c-CV (%)	9.24 ± 1.36	9.05 ± 1.22	9.23 ± 1.32	0.317
Echo data				
LVEF (%)	59.3 ± 4.8	44.3 ± 2.0	34.4 ± 2.6	< 0.001
LAD (mm)	41.9 ± 3.9	41.8 ± 4.3	43.0 ± 4.6	0.001
E/e′	13.0 ± 2.0	12.9 ± 2.2	13.5 ± 2.6	0.005

Data are presented as mean ± SD or number (%) of subjects

*HF* heart failure, *PCI* percutaneous coronary intervention, *CABG* coronary artery bypass graft, *T2DM* type 2 diabetes mellitus, *COPD* chronic obstructive pulmonary disease, *HF* heart failure, *ICD* implantable cardioverter defibrillator, *CRT-P* cardiac resynchronization therapy-pacemaker, *CRT-D* cardiac resynchronization therapy-defibrillator, *ACEI/ARB* angiotensin converting enzyme inhibitor/angiotensin II receptor blocker, *α-GI* alpha-glucosidase inhibitor, *DPP* dipeptidyl peptidase, *NYHA* New York Heart Association functional class, *BP* blood pressure, *eGFR* estimated glomerular filtration rate, *BNP* B-type natriuretic peptides, *HbA1c* hemoglobin A1c, *HbA1c-SD* standard deviation of HbA1c, *HbA1c-CV* coefficient of variation of HbA1c, *LVEF* left ventricular ejection fraction, *LAD* left atrium diameter, *E/e’* mitral Doppler early velocity/mitral annular early velocity

### All-cause mortality

Data on death status was available for all patients. During 42 months of follow-up, 270 (29.9%) patients died from any cause, 75 (25.9%) patients with HFpEF, 35 (26.7%) patients with HFmrEF and 160 (33.3%) patients with HFrEF (HFpEF vs HFrEF: P = 0.031). There were 74 (95% CI 57–91) deaths/1000-patient years in those with HFpEF, 76 (95% CI 54–99)/1000-patient years in those with HFmrEF and 95 (95% CI 84–106)/1000-patient years among those with HFrEF). For multivariable regression analysis in model 1, variables (age, gender, medical history, HF device-therapies, medications, clinical status, laboratory variables and echo data) were entered into the univariate regression analysis, and variables with P < 0.10 [age, HbA1c-SD (high or low), eGFR, ACEI/ARB, beta-blockers, BNP level (tertiles), ischemic HF, NYHA functional class, E/e′ and LVEF (≥ 50, 40–49, < 40%)] and forced inclusion variables that were considered as important predictors of clinical endpoints or associated with HbA1c variability (gender, HbA1c-mean, baseline HbA1c, number of HbA1c measurements) were further entered into the multivariable Cox regression model. The result showed that HbA1c-SD (HR 1.649, 95% CI 1.288–2.110, P ≤ 0.001) as well as other variables (LVEF, BNP and E/e′) were associated with an increased risk of all-cause mortality, ACEI/ARB or beta-blockers therapies were associated with a decreased risk of all-cause mortality (Table 2). When using HbA1c-CV instead of HbA1c-SD in model 2, HbA1c-CV, BNP, LVEF and E/e′ were associated with an increased risk of all-cause mortality, ACEI/ARB or beta-blockers therapies were associated with a decreased risk of all-cause mortality (Table 2).

**Table 2:** Multivariable Cox analysis for all-cause mortality

	HR (model 1)	95% confidence interval	P value	HR (model 2)	95% confidence interval	P value
HbA1c-SD (high, low)	1.649	1.288–2.110	< 0.001	–	–	–
HbA1c-CV (high, low)	–	–	–	1.558	1.216–1.997	< 0.001
E/e′	1.069	1.015–1.125	0.011	1.063	1.010–1.119	0.019
LVEF (≥ 50, 40–49, < 40%)	1.159	1.003–1.340	0.045	1.178	1.019–1.362	0.021
Age	1.014	0.997–1.030	0.109	1.014	0.997–1.031	0.104
eGFR	0.990	0.977–1.003	0.142	0.989	0.976–1.003	0.115
ACEI/ARB	0.705	0.526–0.945	0.019	0.701	0.523–0.940	0.018
Beta-blocker	0.738	0.564–0.965	0.026	0.719	0.549–0.941	0.016
BNP (tertiles)	1.335	1.146–1.555	< 0.001	1.317	1.131–1.534	< 0.001
Ischemic HF	1.076	0.846–1.370	0.550	1.067	0.838–1.359	0.598
NYHA	1.103	0.994–1.287	0.217	1.019	0.949–1.296	0.191
Gender	1.098	0.849–1.421	0.475	1.111	0.859–1.438	0.423
Baseline HbA1c	1.045	0.850–1.285	0.674	1.036	0.843–1.274	0.733
Number of HbA1c measurements	1.034	0.974–1.098	0.277	1.030	0.970–1.094	0.332
HbA1c-mean	1.085	0.895–1.315	0.406	1.191	0.979–1.449	0.080

*HbA1c* hemoglobin A1c, *HbA1c-SD* standard deviation of HbA1c, *HbA1c-CV* coefficient of variation of HbA1c, *E/e′* mitral Doppler early velocity/mitral annular early velocity, *LVEF* left ventricular ejection fraction, *eGFR* estimated glomerular filtration rate, *ACEI/ARB* angiotensin converting enzyme inhibitor/angiotensin II receptor blocker, *BNP* B-type natriuretic peptides, *MI* myocardial infarction, *NYHA* New York Heart Association functional class

### Combined all-cause mortality and heart failure hospitalization

During 42 months of follow-up, 545 patients (60.4%) either died from any cause or were hospitalized for HF, 161 (55.5%) patients with HFpEF, 73 (55.7%) patients with HFmrEF and 311 (64.7%) patients with HFrEF (P = 0.021). For multivariable regression analysis in model 3, variables (age, gender, medical history, HF device-therapies, medications, clinical status, laboratory variables and echo data) were entered into the univariate regression analysis, and variables with P < 0.10 [age, HbA1c-SD (high or low), eGFR, beta-blockers, BNP (tertiles), ischemic HF, NYHA, E/e′ and LVEF (≥ 50, 40–49, < 40%)] and forced inclusion variables (gender, HbA1c-mean, baseline HbA1c, number of HbA1c measurements) were further entered into the multivariable Cox regression model. The result showed that HbA1c-SD (HR 1.485 95% CI 1.251–1.763, P ≤ 0.001) as well as BNP and E/e′ were associated with an increased risk of composite endpoints, beta-blocker therapy was associated with a decreased risk of composite endpoints (Table 3). When using HbA1c-CV instead of HbA1c-SD in model 4, HbA1c-CV, LVEF, BNP and E/e′ were associated with an increased risk of combined endpoints, beta-blocker therapy was associated with a decreased risk of combined endpoints (Table 3).

**Table 3:** Multivariable Cox analysis for composite endpoints

	HR (model 3)	95% confidence interval	P value	HR (model 4)	95% confidence interval	P value
HbA1c-SD (high, low)	1.485	1.251–1.763	< 0.001	–	–	–
HbA1c-CV (high, low)	–	–	–	1.378	1.160–1.638	< 0.001
E/e′	1.045	1.008–1.084	0.018	1.040	01.003–1.079	0.034
LVEF (≥ 50, 40–49, < 40%)	1.093	0.989–1.208	0.081	1.106	1.001–1.222	0.048
age	1.011	0.999–1.022	0.069	1.010	0.999–1.022	0.073
eGFR	0.995	0.985–1.004	0.262	0.994	0.984–1.003	0.184
Beta-blocker	0.810	0.668–0.982	0.032	0.794	0.655–0.964	0.020
ACEI/ARB	1.029	0.827–1.280	0.799	1.018	0.818–1.266	0.873
BNP (tertile)	1.149	1.035–1.276	0.009	1.136	1.017–1.261	0.017
Ischemic HF	1.097	0.925–1.300	0.288	1.095	0.924–1.298	0.296
NYHA	1.075	0.963–1.201	0.199	1.085	0.971–1.212	0.148
Gender	0.916	0.760–1.103	0.354	0.921	0.764–1.109	0.386
Baseline HbA1c	0.994	0.862–1.146	0.933	0.987	0.856–1.137	0.854
Number of HbA1c measurements	1.010	0.968–1.154	0.646	1.007	0.965–1.051	0.739
HbA1c–mean	0.992	0.864–1.140	0.911	1.066	0.926–1.227	0.372

*HbA1c* hemoglobin A1c, *HbA1c-SD* standard deviation of HbA1c, *HbA1c-CV* coefficient of variation of HbA1c, *E/e′* mitral Doppler early velocity/mitral annular early velocity, *LVEF* left ventricular ejection fraction, *eGFR* estimated glomerular filtration rate, *ACEI/ARB* angiotensin converting enzyme inhibitor/angiotensin II receptor blocker, *BNP* B-type natriuretic peptides, *MI* myocardial infarction, *NYHA* New York Heart Association functional class

### HbA1c variability and all-cause mortality/composite endpoints

**Fig. 2:**
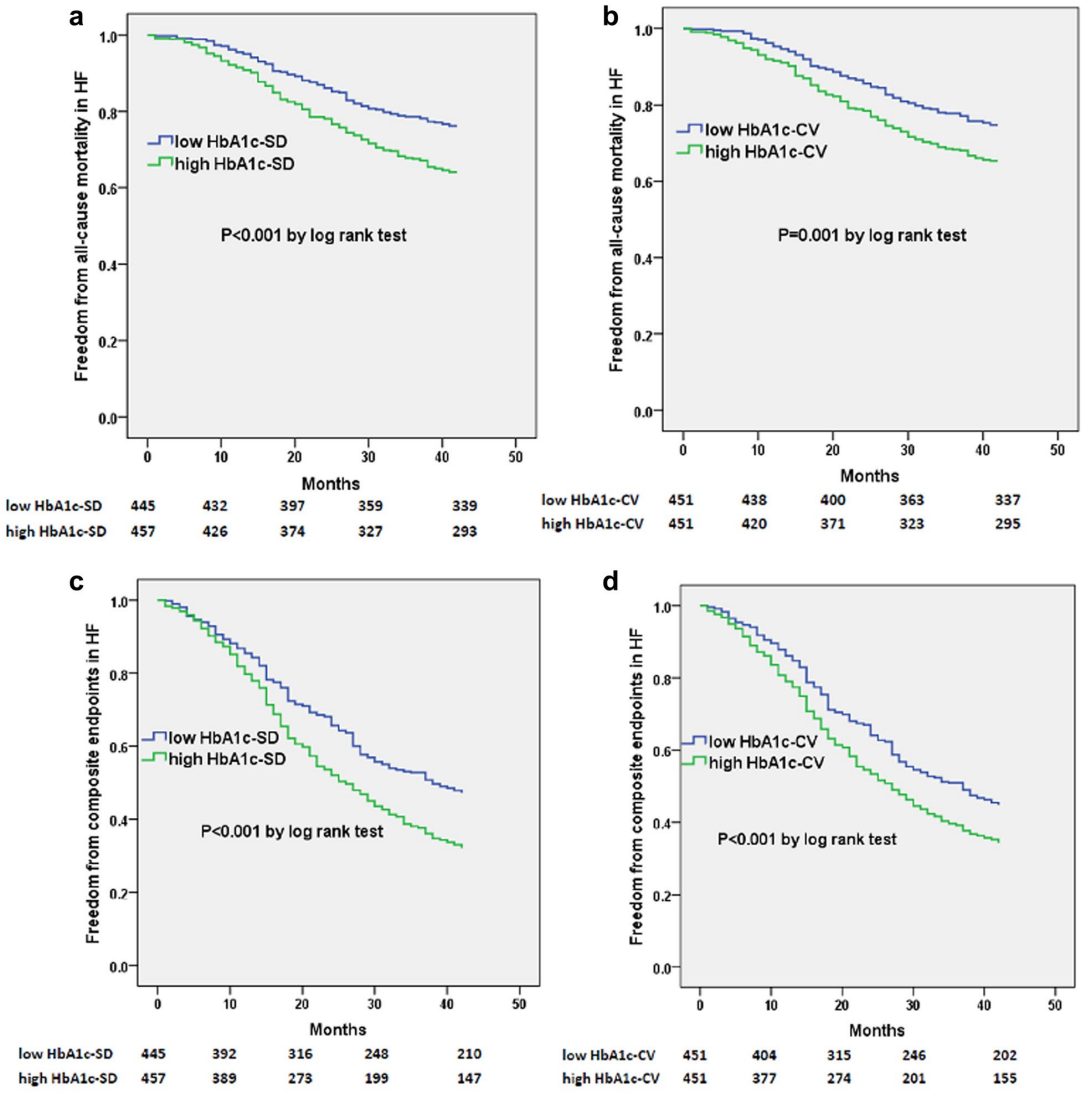
Kaplan–Meier curves of freedom from all-cause mortality (**a**, **b**) and composite endpoints (**c**, **d**) for low and high HbA1c variability after 42-month follow-up in total HF patients. The numbers at the bottom of the figure are “number at risk”

Over a follow-up of 42 months, the percentage of subjects who experienced all-cause mortality or combined endpoints was higher in those with higher glycemic variability group (HbA1c-SD or HbA1c-CV) compared with lower glycemic variability group in total patents as well as subgroups of HFpEF, HFmrEF and HFrEF (see Additional file 1: Table S1). The Kaplan–Meier plot for the occurrence of all-cause mortality or composite endpoints between different HbA1c variability levels were presented in Fig. 2 (HF) and Additional file 2: Figure S1 (HFpEF), Additional file 3: Figure S2 (HFmrEF) and Additional file 4: Figure S3 (HFrEF).

## Discussion

In the present study, we prospectively followed a cohort of HF comorbidity with T2DM over a long-term follow-up period and summarized possible prognostic factors associated with mortality and HF hospitalization. Our current data showed that overall mortality and risk of HF readmission of HFpEF were lower than in HFrEF. Our result further revealed that higher HbA1c variability was associated with elevated all-cause mortality and HF hospitalization and was an independent predictor of all-cause mortality and HF hospitalization, regardless of EF, and a given level of HbA1c variability portended the same risk of death in HFpEF, HFmrEF, and HFrEF. Our study suggested that increase in the long-term glycemic variability might have a detrimental effect on prognosis in patients with HF and T2DM.

Over the years, compared with HFrEF, the clinical outcomes for patients with HFpEF are uncertain and controversial. More than a decade ago, two epidemiological studies reported similar outcomes for patients with these two HF phenotypes [13, 14]. However, a meta-analysis demonstrated that overall those patients with HFpEF had a lower risk of death than patients with HFrEF [15]. The subsequent MAGGIC meta-analysis reported that patients with HFpEF had lower risk of death from any cause compared with those with HFrEF independent of clinical covariates [16]. A recent prospective multi-centre longitudinal study in New Zealand and Singapore showed that the prevalence and mortality were lower in HFpEF than HFrEF [17]. Our present study also indicated that the all-cause mortality and HF hospitalization were lower in HFpEF than HFrEF.

HF, as well as T2DM, is one of the most concerning public health problem worldwide [1, 2]. Diabetic patients with both reduced and preserved EF show increased mortality and morbidity rates compared with patients without diabetes [2, 3]. This increased risk is observed in those diabetic patients of both ischaemic and non-ischaemic origin [2]. In most international guidelines for diabetes management, reducing the blood glucose level, measured by HbA1c, to optimal level is a well-recognized goal to minimize the risk of CVD and death [2]. However, the optimal glucose level has not been well characterized in patients with HF. Some studies showed that higher HbA1c was associated with increased mortality in HF patients [18, 19]. Other data support a paradoxical or J-shaped relationship between HbA1c and clinical outcomes [20, 21], indicating that hypoglycemia might mitigate possible benefits of lower HbA1c. A large cohort study of HF patients with DM showed a U-shaped relationship between HbA1c and mortality, with the lowest risk in patients with moderate glycemic control (HbA1c 7.1–8.0%) [22]. The recent Empagliflozin Cardiovascular Outcome Event Trial in patients with T2DM (EMPA-REG) showed a significant reduction in total mortality, morbidity and risk of HF despite the achieved HbA1c which was 7.8% [23]. Therefore, an important issue that is still unsolved is the target level of HbA1c that should be regarded as optimal in HF patients. Experts recommend relaxed glucose targets among patients with significant comorbidities and an individualized approach based upon the perceived risk of hypoglycemia as well as the potential for adverse sequelae related to hypoglycemia [24, 25]. And hypoglycemia may be particularly concerning in HF patients, due to the predisposition for arrhythmias and ischemic events [26, 27]. In the present study, after categorized at clinically meaningful cut-off values (≤ 7.0, 7–8 and ≥ 8%), HbA1c-mean was not associated with the incidence of all-cause mortality and HF hospitalization. Moreover, HbA1c-mean was also not a risk factor for the incidence of all-cause mortality and HF hospitalization in our enrolled patients after multivariable Cox regression.

Apart from the optimal level, there is an emerging concern about the detrimental effect of glucose fluctuation among diabetic populations [28, 29]. Many studies have indicated that the glycemic variability is a potential predictor for diabetic complications and mortality and might play an important role in clinical risk assessment [28, 29]. Literatures have demonstrated that the short-term effect of higher level of fluctuation in blood glucose is an independent predictor of mortality [4, 5]. Nevertheless, a few studies have investigated the long-term effect of variability in HbA1c [29]. As a whole, most agree that glycemic variability predicts all-cause mortality, fatal or non-fatal CVD in T2DM [30–34]. However, there were opposing reports for these outcomes, the RIACE study revealed that HbA1c variability did not have a major effect on macrovascular complications including coronary or cerebrovascular events, myocardial infarction, or stroke [35]. The discrepancy between previous reports might be explained by differences in the study design and different degree of HbA1c variability, baseline HbA1c level, or ethnicity. Our previous study further revealed that higher HbA1c variability was associated with greater left ventricular diastolic dysfunction and was an independent predictor of new onset of symptomatic HFpEF [8]. Moreover, in patients with T2DM, elevated HbA1c variability was significantly associated with future AF development [9]. In the present study, HbA1c variability was found to be associated with all-cause mortality and HF hospitalization and was an independent predictor of all-cause mortality and HF hospitalization after adjusting for clinical covariates, including EF.

In the pathophysiological rationale, intermittent hyperglycemia rather than chronic hyperglycemia exacerbates the production of reactive oxygen, impairs endothelial function and induces cytokines release and long-lasting epigenetic changes, which will lead to increased risk of CVD and mortality [36]. Besides, hypoglycemia might contribute to the increase in the progression of CVD and mortality though induction of inflammation, blood coagulation abnormality, sympathoadrenal response and endothelial dysfunction [37]. Some studies revealed that glucose fluctuation might be associated with the risk of hypoglycemia [37]. The importance of hypoglycaemia has also been highlighted by the EPHESUS study that found a 38% increased risk of a poorer outcome among patients with hypoglycaemia complicating HF post-myocardial infarction [38]. Rates of severe hypoglycemia are more common among older adults and those with chronic conditions, such as chronic kidney disease, CVD, HF and depression, as well as among those who are on insulin or take secretagogues. Many of the new agents to treat diabetes are less likely to cause hypoglycemia than the older classes of medications [37]. In addition to metformin, glucagon-like peptide-1 (GLP-1) agonists, dipeptidyl peptidase-4 (DPP-4) inhibitors and sodium–glucose cotransporter 2 (SGLT-2) inhibitors are all excellent choices for people who are at risk of hypoglycemia [23, 37, 39, 40]. More interestingly, it has been noted that some interventions (a1-glucosidase inhibitor or SGLT-2 inhibitors) that ameliorate glycemic variability have been found to reduce CVD compared to therapeutics that show less effect on glycemic variability [23, 39, 40].

Predictive values of other parameters in patients HF or diabetes have also been reported. Glycemic variability, as assessed by variability over time in HbA1c, might be an important factor in understanding mortality risk in older people with diabetes [41]. Low 1,5-anhydroglucitol levels, which indicate postprandial hyperglycemia, predict long-term cardiac mortality even in acute coronary syndrome patients with HbA1c levels ≤ 7.0% [42]. Moreover, advanced glycation end-products (AGEs) or soluble receptor of AGE (sRAGE), high-sensitivity troponin T (hs-TnT) and ST2 are also useful markers of HF progression [43, 44].

### Study limitation

The present study should be interpreted in the context of several possible limitations. First, it is an observational cohort study. Potential information biases include changes in the sample examination method with time and differences in the number of HbA1c measurements. In particular, the intervals between HbA1c measurements varied for enrolled patient. And our study merely suggested the association between the long-term glycemic variability and the prognosis of HF, but not the casualty. Second, we did not measure the markers of oxidative stress and endothelial function in the present study, it is widely recognized that glycemic variability causes much more serious oxidative stress and endothelial dysfunction than chronic sustained hyperglycemia. Third, hypoglycemia might be a risk factor for the progression of CVD and mortality, unfortunately, we had not documented the onset of hypoglycemia in our prospective cohort study. Finally, the study participants were from a single center in China, and it is uncertain whether these findings can be generalized to other ethnic groups.

## Conclusions

Overall, the risk of all-cause mortality or combined death and HF hospitalization was lower for HFpEF than HFrEF. HbA1c variability related independently and similarly to risk of all-cause mortality or composite endpoints in the three HF phenotypes. These findings will inform projections of health care needs and the design of therapeutic trials in HF and T2DM around the world.

## Additional files

**Additional file 1: Table S1.** HbA1c variability and the outcome of HF.

**Additional file 2: Figure S1.** Kaplan–Meier curves of freedom from all-cause mortality (A, B) and composite endpoints (C, D) for low and high HbA1c variability after 42-month follow-up in HFpEF. The numbers at the bottom of the figure are “number at risk”.

**Additional file 3: Figure S2.** Kaplan–Meier curves of freedom from all-cause mortality (A, B) and composite endpoints (C, D) for low and high HbA1c variability after 42-month follow-up in HFmrEF. The numbers at the bottom of the figure are “number at risk”.

**Additional file 4: Figure S3.** Kaplan–Meier curves of freedom from all-cause mortality (A, B) and composite endpoints (C, D) for low and high HbA1c variability after 42-month follow-up in HFrEF. The numbers at the bottom of the figure are “number at risk”.

## Abbreviations

HF: heart failure
HFpEF: heart failure with preserved ejection fraction
HFmrEF: heart failure with mid-range ejection fraction
HFrEF: heart failure with reduced ejection fraction
DM: diabetes mellitus
CVD: cardiovascular disease
AF: atrial fibrillation
T2DM: type 2 diabetes mellitus
HbA1c: hemoglobin A1c
eGFR: estimated glomerular filtration rate
HbA1c-SD: standard deviation of HbA1c
HbA1c-CV: coefficient of variation of HbA1c
LV: left ventricular
LVEF: left ventricular ejection fraction
HR: hazard ratios
CIs: confidence intervals
CAD: coronary artery disease
ACEI: angiotensin converting enzyme inhibitor
ARB: angiotensin receptor blocker
NYHA: New York Heart Association functional class
SBP: systolic blood pressure
DBP: diastolic blood pressure
LAD: left atrium diameter
BNP: B-type natriuretic peptide
α-GI: alpha-glucosidase inhibitor
GLP: glucagon-like peptide-1
DPP-4: dipeptidyl peptidase-4
SGLT-2: sodium–glucose cotransporter 2
AGEs: advanced glycation end-products
sRAGE: soluble receptor of AGE
hs-TnT: high-sensitivity troponin T
