# Supplementary material for: Prognostic impact of HbA1c variability on long-term outcomes in patients with heart failure and type 2 diabetes mellitus
Source: Cardiovasc Diabetol. 2018 Jun 30;17:96. doi: 10.1186/s12933-018-0739-3 (PMC6026342; doi:10.1186/s12933-018-0739-3)
Supplement: Supplementary file 1 — Additional file 1: Table S1. HbA1c variability and the outcome of HF. [file 12933_2018_739_MOESM1_ESM.docx]

Table S1 HbA1c variability and the outcome of HF

HbA1c-SD: standard deviation of HbA1c; HbA1c-CV: coefficient of variation of HbA1c; HF: heart failure; HFpEF: heart failure with preserved ejection fraction; HFmEF: heart failure with mid-range ejection fraction; HFrEF: heart failure with reduced ejection fraction.

|  | HbA1c-SD  (mortality ) | | | HbA1c-SD  (Composite events) | | | HbA1c-CV  (mortality ) | | | HbA1c-CV  (Composite events) | | |
| --- | --- | --- | --- | --- | --- | --- | --- | --- | --- | --- | --- | --- |
|  | low | high | P | low | high | P | low | high | P | low | high | P |
| HF  (n=902) | 106/  445 | 164/  457 | <0.001 | 235/  445 | 310/  457 | <0.001 | 114/  451 | 156/  451 | 0.002 | 249/  451 | 296/  451 | 0.001 |
| HFpEF  (n=290) | 30/  145 | 45/  145 | 0.044 | 68/  145 | 93/  145 | 0.003 | 30/  145 | 45/  145 | 0.044 | 71/  145 | 90/  145 | 0.025 |
| HFmrEF  (n=150) | 11/  66 | 24/  65 | 0.009 | 30/  66 | 43/  65 | 0.017 | 12/  65 | 23/  66 | 0.034 | 30/  65 | 43/  66 | 0.029 |
| HFrEF  (n=544) | 64/  239 | 96/  242 | 0.003 | 136/  239 | 175/  242 | <0.001 | 69/  240 | 91/  241 | 0.036 | 144/  240 | 167/  241 | 0.033 |
