# Supplementary figures and images for: Prognostic impact of HbA1c variability on long-term outcomes in patients with heart failure and type 2 diabetes mellitus
Source: Cardiovasc Diabetol. 2018 Jun 30;17:96. doi: 10.1186/s12933-018-0739-3 (PMC6026342; doi:10.1186/s12933-018-0739-3)

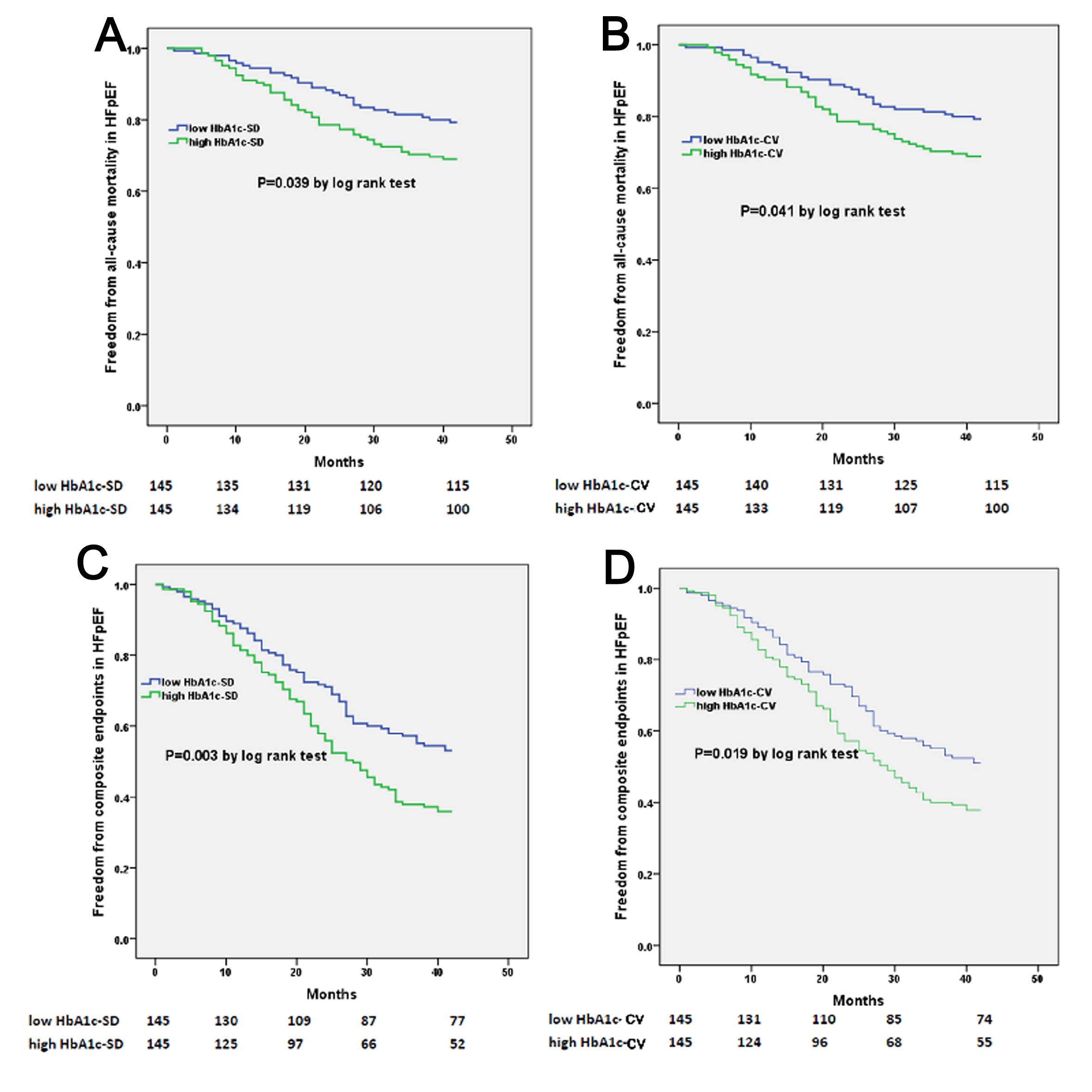

Supplement: Supplementary file 2 — Additional file 2: Figure S1. Kaplan–Meier curves of freedom from all-cause mortality (A, B) and composite endpoints (C, D) for low and high HbA1c variability after 42-month follow-up in HFpEF. The numbers at the bottom of the figure are “number at risk”. [file 12933_2018_739_MOESM2_ESM.tif]

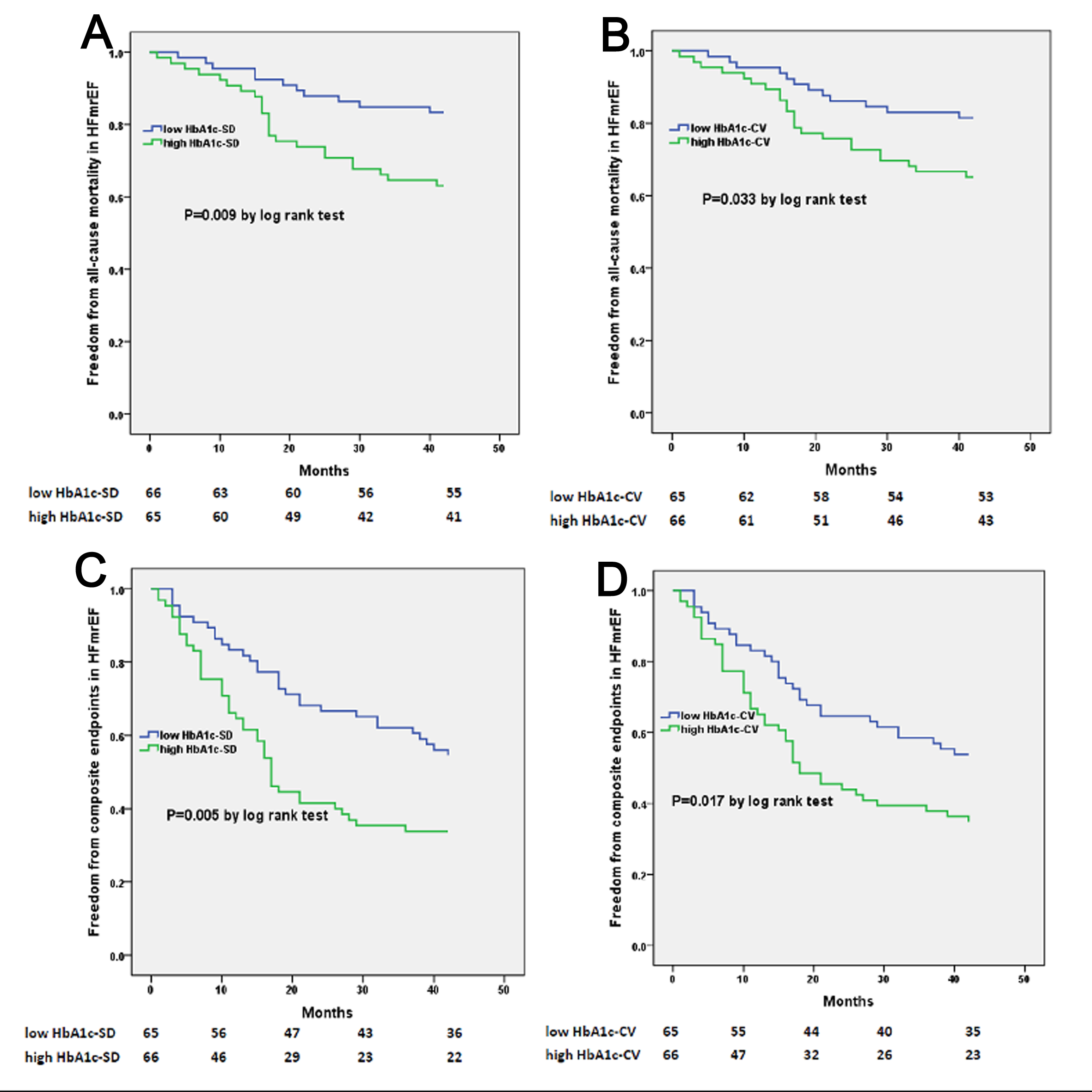

Supplement: Supplementary file 3 — Additional file 3: Figure S2. Kaplan–Meier curves of freedom from all-cause mortality (A, B) and composite endpoints (C, D) for low and high HbA1c variability after 42-month follow-up in HFmrEF. The numbers at the bottom of the figure are “number at risk”. [file 12933_2018_739_MOESM3_ESM.tif]

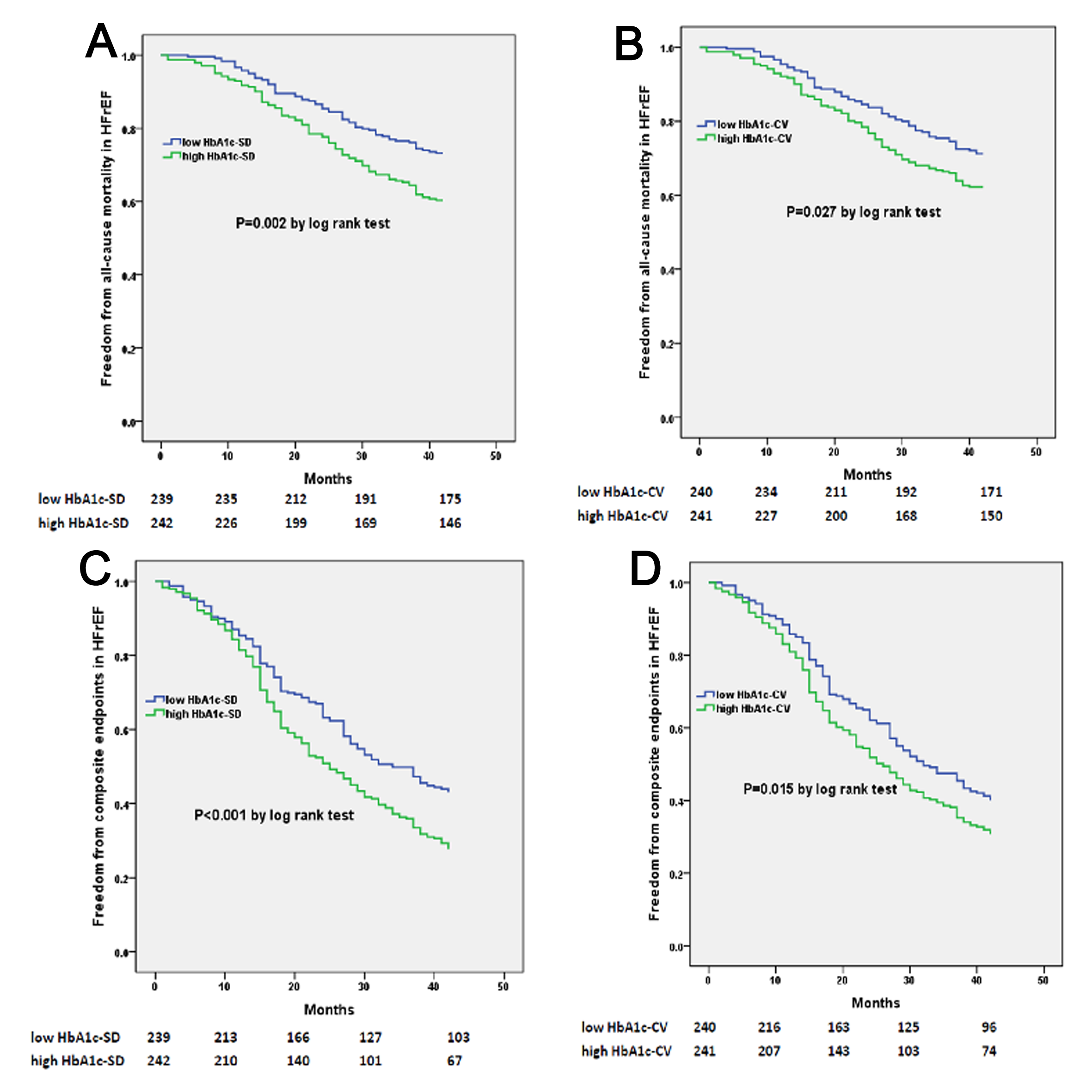

Supplement: Supplementary file 4 — Additional file 4: Figure S3. Kaplan–Meier curves of freedom from all-cause mortality (A, B) and composite endpoints (C, D) for low and high HbA1c variability after 42-month follow-up in HFrEF. The numbers at the bottom of the figure are “number at risk”. [file 12933_2018_739_MOESM4_ESM.tif]
